# National and Provincial Prevalence of Cigarette Smoking in Iran; A Systematic Analysis of 12 Years of STEPS Experience

**DOI:** 10.34172/aim.2023.72

**Published:** 2023-09-01

**Authors:** Elnaz Shahmohamadi, Moein Yousefi, Esmaeil Mohammadi, Ali Ghanbari, Elaheh Shaker, Sina Azadnajafabad, Mohsen Abbasi-Kangevari, Mohammad-Mahdi Rashidi, Negar Rezaei, Sahar Mohammadi Fateh, Elmira Foroutan Mehr, Saral Rahimi, Mohammad Effatpanah, Hamidreza Jamshidi, Farshad Farzadfar

**Affiliations:** ^1^Non-Communicable Diseases Research Center, Endocrinology and Metabolism Research Institute, Tehran University of Medical Sciences, Tehran, Iran; ^2^Endocrinology and Metabolism Research Center, Endocrinology and Metabolism Clinical Sciences Institute, Tehran University of Medical Sciences, Tehran, Iran; ^3^National Center for Health Insurance Research, Tehran, Iran; ^4^Research Institute for Endocrine Sciences, Department of Pharmacology, School of Medicine, Shahid Beheshti University of Medical Sciences, Tehran, Iran

**Keywords:** Epidemiology, Non-communicable disease, Smoking, STEPS

## Abstract

**Background:**

Smoking is a modifiable risk factor for six of the eight leading causes of death. Despite the great burden, there is lack of data regarding the trend of cigarette smoking in Iran. We described the national and provincial prevalence of cigarette smoking and its 12-year time trend utilizing six rounds of Iranian stepwise approach for surveillance of non-communicable disease (STEPS) surveys.

**Methods:**

We gathered data from six STEPS surveys done in 2005, 2007, 2008, 2009, 2011, and 2016 in Iran. To estimate the data of missing years, we used two separate statistical models including the mixed model and spatio-temporal analysis.

**Results:**

The overall prevalence rate of cigarette smoking was 14.65% (12.81‒16.59) in 2005 and 10.63% (9.00‒12.57) in 2016 in Iran. The prevalence of cigarette smoking in 2005 and 2016 was 25.15% (23.18‒27.11) and 19.95% (17.93%‒21.97%) for men and 4.13% (2.43‒6.05) and 1.31% (0.06-3.18) for women, respectively. The prevalence of smoking in different provinces of Iran ranged from 20.73% (19.09‒22.47) to 9.67% (8.24‒11.34) in 2005 and from 15.34% (13.68‒17.12) to 6.41% (5.31‒7.94) in 2016. The overall trend of smoking was downward, which was true for both sexes and all 31 provinces. The declining annual percent change (APC) of the prevalence trend was -2.87% in total population, -9.91% in women, and -2.08% in men from 2005 to 2016.

**Conclusion:**

Although the prevalence of smoking had a decreasing trend in Iran, this trend showed disparities among sexes and provinces and this epidemiological data can be used to modify smoking prevention programs.

## Introduction

 Cigarette smoking is still a serious public health threat and is among the ‘grand’ risk factors for many preventable diseases that cause a high rate of mortality and morbidity.^1,2^ It has been demonstrated that the rate of respiratory cancers and many vascular disorders attributable to cigarette smoking have experienced an upward shift in Iran and many other regions of the globe.^3,4^ Despite the knowledge about its adverse effects, there are 1.1 billion smokers around the world.^5^ As the most preventable cause of death, smoking kills 6 million people annually, of whom 1 million are second or third-hand smokers.^6^ The predictions emphasize that the mortality rate attributable to smoking will be notably higher in 2030.^7^ Although the rate of smoking is decreasing nowadays, the peak of mortality will be three to four decades after the peak of prevalence.^8^ This increase in smoking-related deaths will not be the same in different countries as it will be higher by 9% in high-income countries but in contrast, in low- and middle-income countries, this is expected to be doubled during the same period.^7^

 Life expectancy has been increasing in recent decades due to the improvement of public health care and access. In contrast, smoking has reduced the mean living years by 10 years in consumers.^9^ It is closely associated with various diseases such as lung cancer, chronic obstructive pulmonary disease (COPD), upper aero-digestive cancers, cardiovascular diseases, osteoporosis, and many other conditions; about half of life-long smokers are believed to finally die of cigarette-related disease.^10-12^ Additionally, it is associated with lower quality of life and mental health.^13^ Although smoking and its sequels have long been referred to as a male problem, the prevalence of smoking has increased in women in Western countries.^14^

 The World Health Organization (WHO) has adopted the ‘framework convention on tobacco control (FCTC)” as a set of strategies to prevent adverse effects of smoking.^15^ Surveillance of cigarette usage extent is critical for reaching FCTC. Previous efforts indicated that about 23% of males and 3% of females use tobacco daily in Iran.^16^ There are many other studies about the prevalence of smoking in small communities in Iran but few represent the country as a whole.^17^ There is not a new report to showcase the trend on the scale of the country and provinces.

 We used the Iranian stepwise approach for surveillance of non-communicable disease (STEPS) and aimed to design a model to assess the time trend of cigarette smoking prevalence in Iran from 2005 to 2016 on national and subnational scales.

## Methods and Materials

###  Data Sources 

 Our data was gathered from Iranian STEPS information from 2005 to 2016. STEPS are national cross-sectional surveys that study risk factors of non-communicable diseases in Iran and its regions. The surveys have been done in six non-consecutive years including 2005, 2006, 2007, 2009, 2011, and 2016. In total 221,706 registry records aged more than 18 years were pooled for modeling. Sampling was done in multiple stages that represented adults from each province. For more information, refer to the study protocol and latest STEPS results on smoking prevalence and a pooled analysis.^18-20^ Consent was not required as second-hand de-identified data (STEPS registry data) was utilized. However, informed written consent was obtained from participants in each round of the STEPS study.

###  Definition of Variables 

 The covariates included in the analysis were sex, age, and area of residence. We assessed current cigarette smoking for all STEPS surveys. To determine the prevalence of cigarette smoking among the Iranian population, respondents were asked questions about duration, count, and the number of days per week of smoking. Current cigarette smoking was defined as the use of any number of cigarettes on a daily basis.

###  Statistical Analysis

 Data of six independent non-overlapping STEPS were used. These large datasets were used to achieve a model to predict the gapping years when the survey was not carried out. For this purpose, we had to run rounds of interpolation and extrapolation to yield appropriate predictions for different years. To this end, we used a mixed model to predict smoking prevalence from 2005 to 2016. For missing parts, we considered provinces and years to have random effects on the mixed model. To make a smooth prediction, we used a spatio-temporal model using residuals from the mixed model and modified the residuals using correlations of provinces that were geographically and temporally close to each other for different regions, sex groups, and age groups. Uncertainty interval for prediction was calculated with 1000 times simulation on the spatio-temporal analysis model. Therefore, 0.025 and 0.975 quantiles of all simulations were defined as the lower and upper bound of the uncertainty interval of predictions, respectively.

 To check the validity of the model, we used cross-validation methods and graphical plots. All pooled data were standardized by age and population. Cross-validation was done with nearly 93% of accuracy. R statistical packages (version 3.0.1) and Stata 14 were used to analyze data. The trends were analyzed utilizing annual percent change (APC), calculated by performing a least-squares regression analysis on the natural logarithm of the rates, with the calendar year serving as the independent variable.

## Results

 The overall trend of smoking was declining from 2005 to 2016, as highlighted in Table 1. The prevalence of smoking was 14.65% (12.81‒16.59) in 2005 and 10.63% (9.00‒12.57) in 2016 in the total population. For men, this number changed from 25.15% (23.18‒27.11) to 19.95% (17.93%‒21.97%) and in women from 4.13% (2.43‒6.05) to 1.31% (0.06‒3.18). The declining APC of the prevalence trend was -2.87% in total population: -9.91% in women, and -2.08% in men from 2005 to 2016.

**Table 1:** The Trend of Cigarette Smoking from 2005 to 2016 in Male, Female, and Both Genders

**Year **	**Both Genders (Prevalence/95% CI)**	**Female (Prevalence/95% CI)**	**Male (Prevalence/95% CI)**
2005	14.65 (16.59-12.81)	4.13 (6.05-2.43)	25.15 (27.12-23.18)
2006	14.31 (16.22-12.52)	3.01 (4.88-1.36)	25.62 (27.56-23.68)
2007	14.25 (16.14-12.46)	2.98 (4.83-1.32)	25.52 (27.45-23.59)
2008	13.74 (15.61-11.96)	2.41 (4.23-0.78)	25.07 (26.99-23.14)
2009	13.33 (15.20-11.57)	2.16 (3.98-0.55)	24.50 (26.42-22.58)
2010	13.15 (15.01-11.40)	2.10 (3.90-0.50)	24.21 (26.13-22.31)
2011	13.13 (14.99-11.32)	2.17 (3.99-0.48)	24.08 (25.00-22.17)
2012	12.57 (14.44-10.85)	1.85 (3.67-0.35)	23.29 (25.22-21.36)
2013	12.12 (14.00-10.44)	1.69 (3.53-0.29)	22.54 (24.48-20.60)
2014	11.65 (13.54-10.00)	1.58 (3.42-0.24)	21.72 (23.67-19.75)
2015	11.14 (13.07-9.50)	1.44 (3.31-0.15)	20.84 (22.83-18.85)
2016	10.63 (12.57-9.00)	1.31 (3.18-0.06)	19.95 (21.97-17.93)

**Figure 1:**
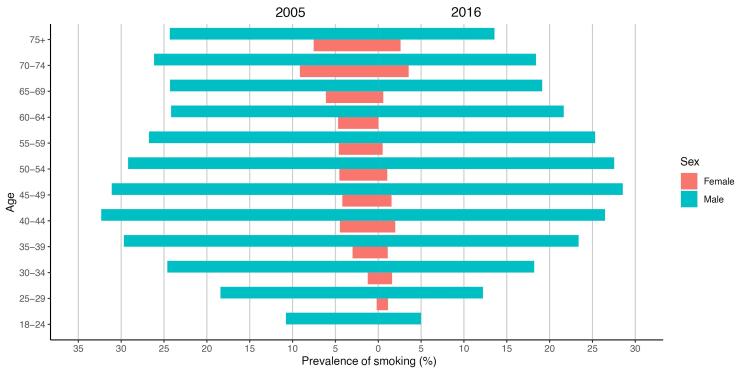
Trend of Smoking from 2005 to 2016 in 31 Provinces of Iran for Males, Females, and Both Sexes

 While the prevalence of cigarette smoking decreased in all age group strata of Iranians older than 18 years, smoking was most prevalent among the 40‒44 years age group in 2005 [18.40, 95% CI: (16.70‒20.10)]; and the 45‒49 years age group in 2016 [15.04, (13.38‒16.82)]. The lowest percentage of cigarette smoking belonged to the youngest age group (18‒24) with 5.39% (CI: 4.53‒6.47) smoker adults in 2005 and 1.95 (CI: 0.96‒2.94) in 2016. More detailed data of the results in age group strata are shown in Table 2. As shown in Figure 1, in both 2005 and 2016, women aged over 75 years were the largest group of cigarette consumers among women; but in 2016, women aged 25‒34 years made up for a higher proportion of smokers compared to 2005.

**Table 2:** National Prevalence of Cigarette Smoking in Different Age Categories

**Age Category**	**2005**	**2006**	**2007**	**2009**	**2011**	**2016**
18-24	5.39 (4.53-6.47)	5.47 (4.62-6.44)	5.73 (4.89-6.67)	5.04 (4.19-5.89)	4.71 (3.86-5.56)	2.50 (1.57-3.42)
25-29	9.30 (8.36-11.00)	9.64(8.72-11.33)	9.90 (8.91-11.60)	9.43 (8.36-11.12)	9.26 (7.84-10.97)	6.68 (5.20-8.52)
30-34	12.92 (11.47-14.63)	13.15 (11.89-14.82)	13.22 (11.92-14.88)	12.80 (11.50-14.48)	12.79 (11.18-14.47)	9.90 (8.20-11.73)
35-39	16.34(14.64-18.03)	16.26 (14.67-18.04)	16.38 (14.70-10.06)	25.59 (14.02-17.27)	15.61 (13.94-17.29)	12.24 (10.78-14.06)
40-44	18.40 (16.70-20.10)	18.47 (16.79-20.15)	18.53 (16.86-20.19)	17.59 (15.93-19.24)	17.56 (15.88-19.24)	14.22 (12.41-16.04)
45-49	17.64 (15.92-19.36)	17.63 (15.92-19.31)	17.61 (15.92-19.30)	17.05 (15.36-18.70)	16.57 (14.90-18.25)	15.04 (13.38-16.82)
50-54	16.86 (15.15-18.59)	16.52 (14.81-18.24)	16.45 (14.77-18.15)	15.93 (14.24-17.63)	15.52 (13.80-17.23)	14.29 (122.87-16.09)
55-59	15.68(13.92-17.44)	15.14 (13.42-16.87)	14.97 (13.228-16.70)	14.34 (12.67-16.07)	14.07 (12.55-15.79)	12.91 (11.77-14.72)
60-64	14.43(12.65-16.20)	13.60(11.8-15.4)	13.48(11.7-15.2)	12.37(10.7-14.1)	12.10(10.55-13.85)	10.82(9.88-12.64)
65-69	15.21(12.84-17.57)	14.28(11.95-16.60)	14.01(11.71-16.33)	12.58(10.33-14.82)	12.71(10.49-14.96)	9.85(8.44-12.11)
70-74	17.65 (14.51-20.80)	16.64(13.56-19.74)	16.29(13.20-19.34)	14.62(11.62-17.67)	14.46(11.48-17.43)	10.97(8.00-13.93)
75 +	15.93 (13.03-18.82)	14.85 (12.01-17.72)	14.42(11.60-17.23)	12.64(9.89-15.41)	12.14(9.41-14.87)	8.07(5.44-10.80)

**Figure 2:**
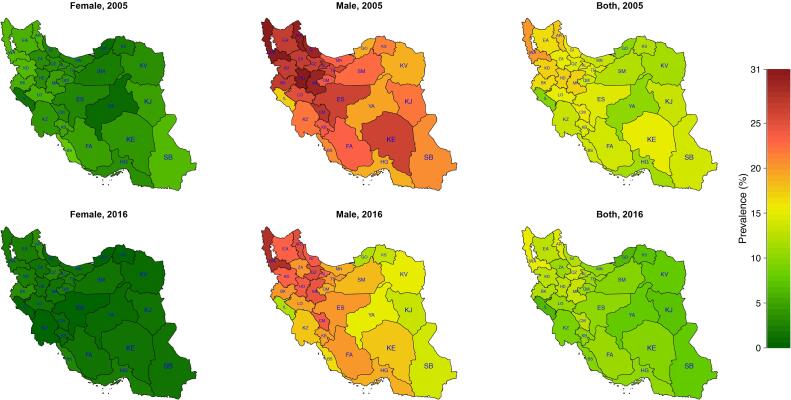
Prevalence of Smoking in 2005 and 2016 for Males, Females, and Both Sexes

**Figure 3:**
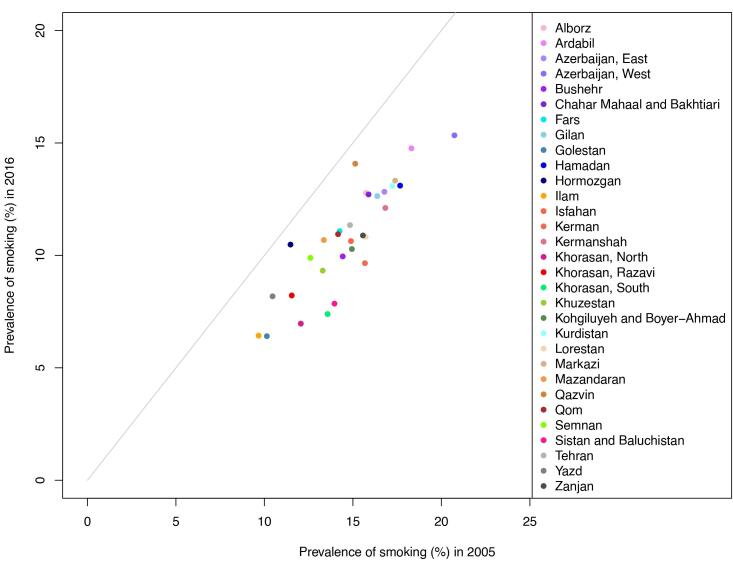
Scatter Plot Showingthe Trend of Smoking Prevalence between 2005 and 2016 in 31 Provinces of Iran

**Figure 4:**
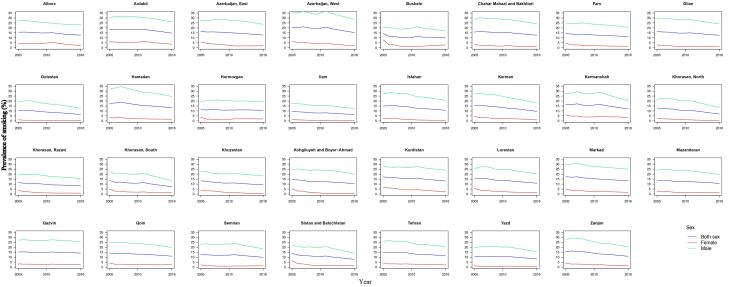
Overall Prevalence of Smoking in 31 Provinces of Iran Based on Sex Strata and Estimates from the Meta-regression Model

 The highest prevalence of smoking was in the northwest region of Iran both in 2005 and 2016 according to Figure 2. Azerbaijan and Ardabil had the highest percentage of smoking with 20.74% (19.09‒22.47) and 18.31% (16.51‒20.08) in 2005 and 15.34% (13.68‒17.12) and 14.76% (13.01‒16.54) in 2016. Ilam and Golestan remained the lowest consumers of cigarettes, with a prevalence of 9.67% (8.24-11.34) in Ilam in 2005 and 6.43% (5.41‒7.58) in 2016, and Golestan with 10.14% (8.63-12.01) smokers in 2005 and 6.41% (5.31‒7.94) in 2016. Although in 2016 twenty out of thirty-one provinces had more than 10% smokers, all of the provinces had a decreasing pattern in these years according to Figure 3. For women, the distribution was different. The highest rate of smoking was in Bushehr 7.94% (6.37‒9.57) and Kurdistan 6.74% (5.07‒8.67) in 2005 and Kermanshah 3.60% (1.95‒5.39) and Ardabil 3.59% (1.87‒5.39) in 2016. However, as shown in Figure 4, the decreasing trend in prevalence of cigarette smoking among women resulted in a converging pattern among provinces over time. Furthermore, women had a lower rate of smokers across all provinces and time periods. A more detailed report of the results is demonstrated in Table 3.

**Table 3:** The Provincial Distribution of Cigarette Smoking Prevalence

**Provinces **	**Prevalence of Smoking (%)-2005 **			**Prevalence of Smoking (%)-2016**		
	**Both Genders**	**Female**	**Male**	**Both Genders **	**Female**	**Male**
Alborz	15.74 (13.52-18.09)	4.04 (1.90-6.37)	27.44 (25.13-29.81)	12.78 (10.67-15.11)	2.30 (0.55-4.56)	23.26 (20.80-25.67)
Ardabil	18.31 (16.51-20.09)	6.22 (4.39-8.04)	30.4 (28.64-32.13)	14.76 (13.01-16.54)	3.59 (1.87-5.39)	25.93 (24.15-27.70)
Azerbaijan, East	16.78 (15.08-18.53)	5.97 (4.30-7.71)	27.59 (25.85-29.35)	12.83 (11.19-14.58)	2.06 (0.59-3.78)	23.59 (21.79-25.39)
Azerbaijan, West	20.74 (19.09-22.47)	6.5 (4.95-8.21)	34.98 (33.23-36.73)	15.34 (13.68-17.12)	2.03 (0.52-3.75)	28.65 (26.84-30.49)
Bushehr	14.43 (12.76-16.10)	7.94 (6.37-9.58)	20.91 (19.16-22.62)	9.95 (8.40-11.68)	2.95 (1.58-4.62)	16.96 (15.22-18.74)
Chahar Mahaal and Bakhtiari	15.89 (14.17-17.64)	3.37 (1.73-5.12)	28.4 (26.61-30.16)	12.71 (11.26-14.58)	1.14 (0.20-2.92)	24.28 (22.32-26.24)
Fars	14.2 (12.58-15.96)	4.58 (3.00-6.24)	23.93 (22.16-25.67)	11.08 (9.51-12.83)	1.62 (0.30-3.32)	20.53 (18.73-22.33)
Gilan	16.38 (14.50-18.30)	2.93 (1.20-4.78)	29.82 (27.81-31.83)	12.64 (11.28-14.29)	1.13 (0.30-2.54)	24.15 (22.25-26.04)
Golestan	10.14 (8.64-12.01)	1.13 (0.19-2.87)	19.15 (17.09-21.15)	6.41 (5.31-7.94)	0.17 (0-1.07)	12.64 (10.61-14.80)
Hamadan	17.67 (16.00- 19.46)	3.33 (1.81-5.11)	32 (30.2-33.81)	13.10 (11.49-14.87)	1.68 (0.39-3.27)	24.53 (22.59-26.46)
Hormozgan	11.47 (9.54-13.44)	3.62 (1.83-5.51)	19.33 (17.25-21.37)	10.48 (8.23-13.11)	1.94 (0.16-4.52)	19.02 (16.30-21.70)
Ilam	9.67 (8.25-11.35)	1.79 (0.73-3.38)	17.56 (15.76-19.31)	6.43 (5.41-7.58)	0.38 (0.05-0.89)	12.48 (10.77-14.26)
Isfahan	14.89 (12.91-17.04)	2.74 (0.91-4.87)	27.04 (24.9-29.21)	10.63 (9.27-12.60)	0.58 (0-2.37)	20.68 (18.54-22.83)
Kerman	15.68 (13.99-17.47)	4.32 (2.71-6.11)	27.05 (25.28-28.82)	9.65 (8.07-11.51)	1.39 (0.23-3.08)	17.92 (15.91-19.94)
Kermanshah	16.83 (15.06-18.70)	6.03 (4.29-7.91)	27.63 (25.83-29.48)	12.11 (10.36-13.91)	3.60 (1.95-5.39)	20.62 (18.77-22.44)
Khorasan, North	12.06 (10.22-13.99)	2.52 (0.92-4.35)	21.6 (19.52-23.63)	6.96 (5.81-8.49)	0.39 (0.02-1.48)	13.54 (11.60-15.51)
Khorasan, Razavi	11.55 (9.93-13.26)	3.67 (2.21-5.32)	19.43 (17.65-21.2)	8.22 (6.98-9.78)	0.90 (0.17-2.16)	15.54 (13.79-17.40)
Khorasan, South	13.57 (11.66-15.64)	4.9 (3.13-6.93)	22.24 (20.2-24.36)	7.39 (5.84-9.35)	1.38 (0.21-3.26)	13.40 (11.47-15.44)
Khuzestan	13.30 (11.67-15.01)	3.82 (2.40-5.42)	22.77 (20.94-24.6)	9.32 (8.16-10.86)	0.48 (0.03-1.72)	18.16 (16.30-20.00)
Kohgiluyeh and Boyer-Ahmad	14.95 (13.02-17.05)	5.23 (3.52-7.33)	24.66 (22.52-26.77)	10.28 (8.93-11.93)	0.68 (0.06-1.92)	19.88 (17.80-21.93)
Kurdistan	17.23 (15.45-19.15)	6.74 (5.08-8.67)	27.72 (25.83-29.63)	13.085 (11.45-14.92)	2.27 (0.79-4.14)	23.90 (22.10-25.69)
Lorestan	15.72 (14.00-17.55)	5.89 (4.26-7.72)	25.55 (23.74-27.38)	10.83 (9.41-12.56)	1.45 (0.41-3.10)	20.20 (18.42-22.02)
Markazi	17.39 (15.61-19.21)	4.94 (3.18-6.78)	29.84 (28.04-31.63)	13.32 (11.87-15.17)	1.69 (0.62-3.46)	24.96 (23.12-26.88)
Mazandaran	13.36 (11.27-15.50)	2.86 (0.90-4.98)	23.86 (21.64-26.02)	10.68 (8.86-12.80)	1.76 (0.43-3.66)	19.61 (17.30-21.95)
Qazvin	15.13 (13.43-16.89)	3.23 (1.60-4.99)	27.03 (25.27-28.79)	14.08-15.85)	2.64 (1.06-4.31)	25.52 (23.66-27.39
Qom	14.16 (11.77-16.77)	4.07 (1.91-6.63)	24.25 (21.64-26.91)	10.94 (8.67-13.55)	2.38 (0.51-4.93)	19.50 (16.83-22.17)
Semnan	12.60 (10.96-14.42)	2.22 (0.79-3.94)	22.98 (21.12-24.9)	9.89 (8.55-11.44)	1.32 (0.57-2.53)	18.46 (16.53-20.35)
Sistan and Baluchistan	13.96 (11.68-16.31)	6.45 (4.28-8.67)	21.47 (19.07-23.95)	7.85 (6.28-9.78)	1.63 (0.71-3.25)	14.08 (11.84-16.32)
Tehran	14.84 (12.36-17.40)	3.67 (1.37-6.22)	26 (23.35-28.58)	11.35 (9.26-13.83)	2.20 (0.50-4.64)	20.49 (18.02-23.02)
Yazd	10.46 (9.03-12.32)	1.25 (0.39-2.96)	19.67 (17.67-21.68)	8.18 (6.89-9.97)	0.73 (0.07-2.35)	15.63 (13.70-17.59)
Zanjan	15.57 (13.91-17.32)	3.74 (2.26-5.43)	27.39 (25.57-29.2)	10.89 (9.49-12.57)	1.31 (0.32-2.87)	20.47 (18.65-22.28)

## Discussion

 This study provides a trend for cigarette smoking from 2005 to 2016 and shows a total reduction of 5% in the prevalence of smokers in these years. Additionally, it has been emphasized that the decreasing trend is more pronounced in women compared to their male counterparts and showed a converging pattern among provinces. Nevertheless, in almost two thirds of the provinces, still more than 10% of the population remained smokers.

 The reduced rate of smoking in Iran can be due to improvements in population awareness about the harmful effects of smoking, banning cigarette advertising, advertising against smoking in media and even on the pockets of cigarettes, and the prohibition of smoking in public places.^21-23^ Iran’s Comprehensive National Tobacco Control Act was passed in October 2005, which included policies to provide smoking cessation services in all health clinics for anyone wishing to quit smoking.^24^ Moreover, recently, attempts were made by the Iranian healthcare system to restrict the use of this substance.^25^ Nowadays, people are more educated, and it can be one of the main reasons for the declining pattern of smoking, as it was the most important factor affecting the prevalence of smoking in Poland in 2019.^26^ One of the effective ways of lowering cigarette consumption is increasing the tax.^27^ For example, each 10% increase in cigarette price lowers consumption by 5%.^28^ This method should be implemented with caution because of the higher prevalence of smoking in low-income populations.

 The WHO estimated that 20.2% of people aged more than 15 years were smokers in 2015 and indicated a 6.7% reduction in smoking globally since 2000.^29^ In that report, it has been claimed that in 2015, the prevalence of smoking in Iran was 11.14% which is nearly half the global rate.^29^ In our study, the declining average percent change (APC) of the prevalence trend was -2.87% from 2005 to 2016. The smoking prevalence among individuals aged 15 to 69 years decreased overall from 14.6% to 11.7% (APC: -2.73%) from 1991 to 1999 in Iran, showing that the decreasing trend has almost remained the same in the last three decades.^30,31^ According to the different definitions of smokers, the prevalence of tobacco smoking could be different.^32^ In any case, even some STEPS studies and pooled analyses showed an otherwise trend of smoking in Iran in recent years, which is amenable to the methods of analysis that were used and their definition.^31,33^ In contrast to Iran, Taiwan reported little change in prevalence between 2001 and 2005, but after 2009 a dramatic decrease pattern was achieved.^34^ Also, Brazil showed a decreasing pattern in both sexes, all age groups (except between 55 and 64 years) and education levels from 2006 to 2013.^35^ This downward trend was seen in the prevalence of overall tobacco use, heavy smokers and passive smokers at home. Studies demonstrate that without the implementation of further policies and interventions, the world will experience a slow reduction in smoking prevalence. Nonetheless, for better results, we should consider measures such as mass-media campaigns, an increase in tobacco taxes, public smoking prohibition, special courses in schools, and restriction of marketing.^36^ These methods should be considered according to the demographic and provincial cultures and backgrounds.

 In our study, smoking prevalence was 15-times higher in men than women in 2019. This difference is also seen in other countries of the Middle East like Kuwait, Saudi Arabia, and Oman.^37-39^ Compared to the global prevalence of 32.6% for men and 6.5% for women in 2020, Iran shows a higher gender gap.^40^ An interesting finding of the current investigation was that women aged more than 75 years were the largest group of consumers among women in 2005 and 2016; but nowadays, women aged 25‒34 years are more interested in smoking. However, it should be reminded that due to the lower sample size in extreme subgroups of STEPS, e.g., older women responding ‘Yes’ to questions related to smoking, the model loses its power and the results should be taken cautiously with consideration of errors.^41,42^ Even with the total reduction of smoking in women in this study, younger women smoke more cigarettes than 12 years ago. This can be due to the empowerment of women and changes in traditional sex roles in recent years in Iran.^43^

 Despite various levels of smoking in different provinces, the investigation of the subnational distribution of smoking prevalence revealed the same decreasing trend across the country, with West Azerbaijan and Ardabil having the highest and Golestan and Ilam having the lowest proportion of cigarettes consumers throughout the study period. The northwest and western regions of Iran had a higher prevalence of smoking. The high prevalence of smoking in border provinces like Ardebil and West Azerbaijan shares similarities with neighboring countries like Azerbaijan and Turkey, which might be a result of cultural and ethnic resemblance of people in these areas.^44^ Other studies also showed that about one-fourth of the male population in northwestern Iran smoke cigarettes on a daily basis.^32^ These are the regions that showed a higher incidence of lung cancer, as well.^45^ These findings demonstrate the need for different interventions and higher attention to reducing the smoking prevalence in these regions. The distribution of smoking prevalence was not similar between sexes, indicating different levels of the stigma of smoking for females across provinces.

 Low-income and less-educated populations were more likely to be smokers in Iran. These findings had similarities with results from other countries such as the United States.^46^ Despite our findings of the low prevalence of smoking among those aged 18‒24 years, and more educated people, the results of a systematic review done between 2001 and 2011 in Iranian university students showed a prevalence of 19.81% (17.7‒21.9) among men and 2.2% (1.4‒3.02) among women.^47^ Also, this meta-analysis showed different frequencies of smoking in different studies. This difference can be due to the different years of the studies, stigma, subject variation, location, and other factors.

 Smoking is the main risk factor for many cancers and cessation is the easiest and readiest preventive strategy for controlling this great burden; however, countering the manufacturing companies considering the extent of their revenue is not anywhere close to easy.^2,4,48^ COVID-19, the recent pandemic that affected the lives of millions around the world, had a higher toll in smokers in terms of severity and mortality.^49^ In Iran, although the rate of smoking is steadily decreasing, the number of lung and airway neoplasms did not follow the same path.^50^ This remark can be related to the fact that, as an indolent condition, there is a great temporal delay between interventions [i.e., efforts to reduce smoking] and the outcomes [e.g., respiratory cancer incidence]. Hence, continuous examination of the situation is a *sine qua non* of any health-care and observatory system. Moreover, in rhetoric or reality, we are still dealing with the consequences of the massive use of tobacco and other irritating compounds of lining mucosa as lung cancer is still among the leading neoplasm of the human body. Health authorities should be prepared for this mega burden, and take into trial the effectiveness of acts, and the access of entangled patients to health-system goods and quality care.^2,51^

 The inclusion of several years of STEPs observations and statistical models and meta-regression analysis for the prediction of missing periods are among the strengths of this effort. In this survey, we tried to model and estimate the portion of (sub-)populations that have consumed cigarettes in a daily fashion, pooling light users with heavy smokers. Moreover, other unconventional nicotine and tobaccos are major market holders, especially hookah in Iran and Arab neighbors. Future STEPS and other population-wide studies can delineate the influence of COVID-19 on smoking. Efforts to delineate the attributable risk of smoking to other capstone disorders such as vascular disorders and cancers, as well as death and disability are encouraged in future studies.^41,42^

## Limitations and Future Perspective

 This study has several limitations that should be considered. Data used in this study were gathered through face-to-face interviews without further examinations. Data were collected by a wide number of researchers in different populations of six rounds of STEPS surveys, which could have resulted in heterogeneity in data entry and validation. Moreover, similar to other modeling and estimate techniques, the spatio-temporal model used in this study is susceptible to statistical flaws and may be different from real-world data. Because of the stigma of smoking in Iran, especially among women, the prevalence may be underestimated. Also, this stigma is variable in different regions of Iran because of cultural differences. Although the underestimation is believed to be widespread among different age and sex subgroups and has minimal effects on the model. We did not include children in this study, while smoking among students is an important issue. The main strength of this study is its great sample size investigating national and sub-national levels of Iran. On the other hand, the inability to perform annual investigations on the status of risk factors and disorders in the level of population is a shortcoming of most health care systems.

## Conclusion

 In conclusion,population-wide studies are a fundamental part of any healthcare system and are required to understand the situation around a country considering major disorders and risk factors.^52^ Consumers of cigarettes have become less and less in recent years in both sexes in Iran. Therefore, health policies and programs should mainly consider males and younger women. Every sex, age group, and province may need different strategies. According to the gap between the peak of cigarette smoking and related disease culmination, the health system should be prepared for the negative outcomes of smoking.
